# First Bristol Radiology Course, November 1989

**Published:** 1990-12

**Authors:** M.J. Weston, D. Glew, A. Tottle, D.J. Grier


### 1989 Bristol Cardiac Radiology Course

The first Bristol Cardiac Radiology Course was held on 18th November 1989 at the Edward Jenner Centre. The papers presented are summarised in the following report.

**Adult Chest X-ray Interpretation, Dr. Gishen (King's College, London)** He stressed the importance of lower lobe arterial constriction combined with upper lobe blood diversion in the assessment of left ventricular failure, and went on to discuss the 'tubology' which should be recognised, and checked, on post-operative films.

**Paediatric Chest X-ray Interpretation, Dr. Raphael (National Heart Hospital)** The neonatal chest radiograph should allow the Radiologist to answer two questions: Can pulmonary disease be excluded in a sick neonate? Can congenital heart disease be confirmed? On occasions, a specific cardiac diagnosis can be suggested, but these are limited by the slow rate at which characteristic cardiac adaption occurs, by the presence of the thymus and by the potential complexity of the cardiac lesions.

**Nuclear Cardiac Imaging, Dr. Wilde (Bristol)** Reversible defects in myocardial perfusion can be a specific, if insensitive test for coronary artery disease; infarct imaging can be confirmatory when routine tests are inconclusive and ventriculography allows the quantitation of myocardial damage and the assessment of treatment. Uses of nuclear cardiology may be extended by the introduction of radiolabelled monoclonal antibodies and Technetium—99 m radiopharmaceuticals. cals.

**Echocardiography—Technical Developments, Professor Wells (Bristol)** The opening session was concluded with a lucid summary of the major technical advances which have revolutionised echocardiography in the last decade. These from noise reduction and image storage to duplex scanning, colour flow doppler and transoesophageal probes.

**Echocardiography—Imaging and Doppler in Adults, Dr. Wilde (Bristol)** Examples were show of how the improved resolution now available allows easier diagnosis, and has enabled echocardiography to become the major part of cardia diagnosis. The various advantages and disadvantages of pulsed and continuous wave Doppler as well as colour flow were illustrated. In the assessment of valve disease, cardiac catheterisation has been reduced to a pre-surgical technique and in some cases may not be needed at all.

**Echocardiography—Transoesophageal, Dr. Ettles (Bristol)** The clarity of the images was impressive, so much so that many extra small abnormalities were seen whose significance was not always certain. Transoesophageal echocardiography was advocated in intraoperative patients, patients on intensive care wards and in an outpatient setting where there were no clear praecordial ultrasound windows. Sedation is not always necessary. In those that do not require sedation the examination is often quicker than the conventional praecordial technique as a good view is always obtained. Further work is being done on transoesophageal colour flow.

**Echocardiography—Paediatric, Dr. Martin (Bristol Children's Hospital)** Children have numerous good echocardiographic windows, allowing a systematic sequential analysis of the heart. This comes under seven headings: What is the atrial situs, the position of the cardiac apex, the atrioventricular connection and the ventriculo arterial connection? Are there any associated structural anomalies or abnormalities of function? The use of Doppler for quantification and clarification.

**Cardiac Computerised Tomography, Dr. Rees (Killingbeck Hospital, Leeds)** After lunch Dr. Rees gave an interesting talk on cine Computerised Tomography from his experience in the United States. Due to the X-rays being produced by electron beam deviation rather than conventional tube rotation the scan time per slice is reduced to 50 milliseconds. This has great advantages for children, reducing the need for general anaesthesia, and for the extremely ill. Post contrast medium scans shown on a cine loop give excellent visualisation of the flow of contrast medium through the heart, and have been used successfully to study CABG patency, LV volume, LV mass and stroke volume estimates.

**Cardiac Magnetic Resonance Imaging (MRI), Dr. Hartnell** The importance of MRI in complicated cardiac abnormalities was shown, particularly as an adjunct to echocardiography in congenital heart disease. At present only 1.5% of scans performed on the Bristol MRI scanner are for cardiac diseases. More cardiac MRI scans have to be performed if experience is to be gained and software developed to enable more conditions to be examined using MRI. These include graft patency, acute aortic dissection, quantification of shunts and gradients and ischaemic heart disease screening. MRI is now quite cost effective, each scan costing only £110 for running expenses.

**Cardiac Aspects of Contrast Media, Dr. Dawson (Hammersmith)** The cardiovascular effects of contrast media were summarised. His must be the quote of the meeting, "A drug is an agent which when injected into a guinea pig produces a scientific paper". Dr. Gishen and Dr. Raphael concluded the first afternoon session with a practical and clear demonstration of coronary anatomy, and with advice on both adult and paediatric angiography.

**Coronary Angioplasty, Dr. Ruttley (University Hospital Wales)** The pioneering work of Dotter (peripheral vessel dilation using bougies) and Gruntzig (balloon angioplasty) which enabled the development of coronary angioplasty (CA) was described. The technique of CA was illustrated with allusion to the increasing number of patients now considered suitable for it. There is a necessity for facilities for immediate cardiac surgery because of the complications that can occur. Though most centres performing the technique achieve a success rate of 80–90% per vessel, up to one third of these may restenose within six months. As a result, angioplasty will delay but not replace the need for surgery in a significant number of patients. There is therefore considerable scope for improvement.

**Valvoplasty, Dr. Hartnell** Valvoplasty of a stenosed but otherwise normal pulmonary valve is now the treatment of choice. Aortic valvoplasty has initially good symptomatic and objective results, though the latter may not be sustained. It is appropriate in patients who are unfit for surgery by virtue of other medical conditions or severe left ventricular dysfunction. Mitral valvoplasty for congenital or acquired stenosis is valuable in those unfit for or refusing surgery. It may delay surgery for those who also have aortic valve disease, enabling one combined operation rather than two separate valve replacements. It may eventually replace mitral valvotomy.

**Future Developments in Interventional Cardiac Radiology. Dr. Rees** The potential of angioscopy was discussed with the help of videos demonstrating normal, diseased and treated arteries. The results of angioplasty can be directly inspected, and use of angioscopy may enable avoidance of intimal dissection. The use of lasers in peripheral and coronary angioplasty has met with variable success, as have various mechanical devices designed to cross lengths of arterial occlusion. Endovascular ultrasound is another largely unexplored area which will receive more attention in years to come.

The whole course was well presented with speakers keeping to time and high quality slides being shown. There were several exhibits on view, including examples of video and cine film. It is to be hoped that this valuable course will be repeated regularly.

M.J. WESTON, D. GLEW, A. TOTTLE, D.J. GRIER

